# Practicality of the My Baby Now App for Fathers by Fathers: Qualitative Case Study

**DOI:** 10.2196/64171

**Published:** 2025-02-21

**Authors:** Mathew Gaynor, Kylie Hesketh, Kidane Gebremariam, Karen Wynter, Rachel Laws

**Affiliations:** 1 Institute for Physical Activity and Nutrition, School of Exercise and Nutrition Science Deakin University Geelong Australia; 2 Centre of Research Excellence in the Early Prevention of Obesity in Childhood National Health and Medical Research Council Melbourne Australia; 3 Centre for Women’s and Children’s Mental Health, Department of Psychiatry Monash University Melbourne Australia; 4 School of Nursing and Midwifery Deakin University Melbourne Australia

**Keywords:** fathers, parenting resources, health promotion, My Baby Now, MBN, app, mobile phone

## Abstract

**Background:**

Evolving societal trends are resulting in fathers having an increasing influence on the health-related behaviors that children develop. Research shows that most fathers are committed to their role and when equipped with knowledge, can have a positive impact on their child’s health. However, parenting resources typically target mothers, with fathers being excluded. While evolving mobile phone technology provides an efficient means for delivering parenting resources, many fathers find that mobile health (mHealth) technology does not provide material they can engage with.

**Objective:**

This study aimed to explore how to make parenting apps more engaging and useful for fathers using an existing parenting mHealth resource, the My Baby Now app, as a case study.

**Methods:**

A total of 14 purposefully selected, Australian fathers of 7 months to 5-year-old children took part in a qualitative study, comprising either focus groups or interviews. Recorded focus groups and interviews were transcribed verbatim, then coded using a combination of deductive and inductive methods. Reflexive thematic analysis was undertaken to identify patterns and themes.

**Results:**

Current parenting apps provide parenting information that can be unappealing for fathers. To improve paternal engagement with mHealth resources, fathers highlighted the need for father specific information, with an increase in positive imagery and positive descriptions of fathers in their parenting role. There should be father-exclusive domains such as forums, and also push notifications to provide positive reinforcement and encouragement for fathers.

**Conclusions:**

mHealth has the capacity to deliver information to fathers when needed. This reduces the risk of paternal frustration and disengagement from parenting. Further benefit will be gained by research to understand possible differences in mHealth app usage by fathers of differing socioeconomic position, cultural backgrounds, and family status, such as single fathers and same-sex couples.

## Introduction

Parents have a central role in shaping children’s long-term health behavior by the behaviors they promote and role model [1-5]. Changing societal trends of increasing rates of maternal participation in the workforce have resulted in child rearing activities in the home environment becoming increasingly shared with fathers [6,7]. Fathers are, thereby having an increasing influence on the health-related behaviors that children develop [8]. Emerging research shows that when fathers are equipped with skills and knowledge, they can have a positive influence on the dietary and physical activity behaviors of their children [9,10]. However, many fathers lack knowledge and confidence about how to do engage with their children [10]. Multiple barriers that exist to gain skills and knowledge, including a lack of father-focused support, resources, and services are exacerbating this problem for fathers [3,11,12], lack of trained staff specifically to work with fathers [13], differing cultural beliefs toward fathers’ roles [8], and a lack of father-specific, best practice guidelines [9,14]. In addition, paternal involvement in traditional parenting education programs has been low owing to fathers’ full-time employment and not being available for parenting programs during traditional office hours [15], perceived attitudes of stoicism and self-reliance in fathers [16], and programs failing to engage fathers [17,18].

The proliferation of mobile phone usage may present an avenue to conveniently deliver relevant parenting resources and information to fathers [13]. Mobile phone ownership is pervasive in high income countries at over 90% [19] and increasing rapidly in low- and middle-income nations [20-22]. Through their personal mobile devices, parents now have at their disposal, a multitude of resources and information that can be accessed, including websites, social media and apps [23]. International trends for mobile devices being used to seek parenting information are increasingly becoming apparent, where for instance, recent Canadian research shows that 81% of parents rated their smart phone as a more important parenting resource than books (56%) [24].

International parenting research has shown positive results in providing perinatal health information to mothers through their mobile phone, whereby mothers found that they learned information in the perinatal period they otherwise would not have known without the use of texts to their personal phone [25]. Also, recent evidence in Australia and Africa has demonstrated increases in both maternal and paternal knowledge and confidence to breastfeed through the provision of a parenting app during the perinatal period [26,27]. Despite the convenience of parenting apps however, fathers have invariably experienced frustration in their use as a parenting resource, with information being predominantly mother-centric, and some even trivializing the roles of the fathers [28]. However, of the limited evidence of apps specifically targeting and providing information for fathers, positive experiences have been noted by fathers of being provided with convenient and nonjudgemental support [29,30]. Similarly, the Australian Milk Man app used such strategies as push notifications and social connectivity with other fathers to engage fathers with breastfeeding information [31]. This provided the impetus for increased conversations with partners about the benefits and facilitation of breastfeeding.

At present, despite research indicating that mobile health (mHealth) resources and apps are an effective tool for parents, there is a paucity of evidence examining the acceptability, usefulness, and effectiveness of such resources for fathers [32]. Evaluations of the limited number of apps for fathers (ie, Milk Man and mDad) have provided some evidence of the benefits of using these resources for support and guidance of fathers in limited areas of parenting, such as breastfeeding. However, there is a need to consult directly with fathers to gain greater understanding of their specific needs and preferences for mHealth resources promoting positive dietary and physical activity behaviors in young children. This will inform understanding of how mHealth parenting resources can be made more engaging for fathers to use. To our knowledge, no app exists exclusively for fathers to promote positive dietary and active play/physical activity behaviors in children aged 5 years or younger. Therefore, the aim of this study is to explore how to make parenting apps more engaging and useful for fathers using an existing app (My Baby Now) as a case study.

## Methods

### Study Context- Use of the My Baby Now App

This study used the My Baby Now (MBN) app as an example of an existing parenting app for fathers to provide feedback on. This app was chosen as it is currently being made available to parents as part of the roll out of the established Infant Feeding and Active Play and Nutrition (INFANT) program, which is a broader evidence-based group program that focuses on providing anticipatory guidance on feeding and play from birth to 18 months of age across Victoria, Australia [33]. The MBN app provides evidence-based information and support on infant feeding and active play from pregnancy to 18 months of age, in line with Australian Infant Feeding Guidelines and 24-hour Movement Guidelines for Early Years [34,35]. Presented as a parenting app with family imagery and language throughout, the MBN app has a strong focus on developing parenting confidence and skills. The app includes topics on feeding (breast, formula, mixed feeding and introduction to solids, and recipes) sleep and feed patterns, play, parental well-being, and dental care. Users receive 3 push notifications per week tailored to their child’s stage of development and feeding mode (breast, formula, or mixed feeding). A facilitated forum to share experiences with other users is provided along with activities such as goal setting and quizzes to provide tailored feedback in areas such as feeding practices [26]. The app is informed by extensive formative research with mothers [36] and practitioners [37] and a feasibility study of an earlier version of the app, “Growing healthy” [38]. The app has been found to be acceptable and useful to mothers; however, it is less well used by fathers with the vast majority of participants being mothers [26].

### Study Design

This study used qualitative inquiry in focus groups and one-on-one interviews to gain an understanding of fathers’ views and experiences in using mHealth resources and to obtain fathers feedback on the MBN app. The overall methodology of this study was guided by a pragmatic research paradigm, which provides the flexibility to apply research methods best suited to answering a research problem [39]. In our study, qualitative enquiry [40] with thematic analysis [41], was deemed most suitable. In allowing for different worldviews of researchers and participants to be represented [42], the authors recognize that the data will be socially constructed.

### Recruitment

All participants in this study were recruited from a larger, nationwide Australian study [9], that surveyed fathers about their perceived role, self-efficacy and support needs in promoting positive dietary and physical activity behaviors in early childhood. The survey was open to fathers or expectant fathers (aged 18 years or older) with children aged 5 years or younger residing in Australia, with sufficient fluency in English to complete the survey. At the completion of the survey, participants were asked if they would be interested in participating in further research. Email invitations were sent to those who had expressed interest in further research to invite them to participate in the focus groups and interviews. If participants did not respond to the initial email, 2 further reminder emails, at 1-week interval were sent. Once the participants replied to the original or a reminder email, confirmatory emails were sent with details of the zoom meeting and instructions on how to download the MBN app.

Depending on their availability or preference, participants were offered the choice of being in a focus group or having a one-on-one interview. Focus groups and interviews occurred 1-2 weeks after the confirmatory email was sent.

### Data Collection

As part of the larger survey study [9], sociodemographic characteristics of participants were collected including, fathers’ age, level of education, employment status, marital status, location, country of birth, languages spoken at home, number of children and children’s age. Informed by the end user version of the mobile rating scale (uMars), which provides a reliable method to assess the quality of mHealth apps [43], a broad, semistructured focus groups and interview guide was used for this study (Multimedia Appendix 1). The guide was developed to explore fathers’ perceptions of the appeal and usefulness of the solids, feeding, and play sections of the MBN app. After using the MBN app in the 1-2 weeks before the focus groups and interview, fathers provided feedback on their experiences seeking information in MBN, the functionality and aesthetics of MBN, their engagement with MBN, and their opinions of the information within the MBN app. The guide was reviewed by 2 researchers (MG and RL), and minor amendments were subsequently made to the wording of some questions to avoid using leading questions and to improve clarity.

In the confirmation email which participants received before their focus group, they were asked to download the MBN app to their phone before the meeting and were encouraged to explore the features of the app. Participants were advised that during their focus groups and interview, the solids and play sections of the app would be discussed in detail. All clients reported that they were able to download the app before their focus groups and interview as directed. During the focus groups and interviewss, participants used Menti (poll maker) to vote on the usefulness of a sample of 5 MBN app push notifications by selecting one of 5 options, very helpful, helpful, neutral, unhelpful, and very unhelpful. The Menti app enables multiple users to share knowledge and real-time feedback in meetings [44]. Participants voting provided impetus for further discussion on their subjective opinions on the positive or negative aspects of the notification and how it may be improved. The format for rating the push notifications was the same for both focus groups and interviews.

The lead author (MG) conducted the focus groups and interviews between April and May 2023, and these were recorded with the permission of the participants and transcribed verbatim. Each focus group had a primary facilitator, and an observer (KG), who completed reflexive notes.

### Data Analysis

All focus groups and interviews were transcribed by an online platform (zoom) and then manually checked for accuracy by the interviewer. To minimize participatory burden on fathers, transcripts were not returned to participants for review. The interviewer (MG) and observer (KG) took notes throughout the interview and these notes were reviewed with the interview transcripts.

Reflexive thematic analysis was used to identify patterns and themes. Phases of analysis were based on Braun and Clarke’s [41] 6-step method and were conducted by the first author. Initially, the data were coded deductively using uMars as a framework, and then inductive coding was used for data that did not align with uMars domains [45]. After initial coding by the first author, the coding framework was discussed with the research team with a sample of 2 transcripts, resulting in minor refinements. NVivo 11 (Lumivero) was used for coding and retrieval of data [46].

### Researcher Reflexivity and Credibility

Qualitative research relies on nuanced judgements that require researcher reflexivity, to account for how subjectivity shapes their inquiry [47]. MG is a PhD candidate and a registered psychologist and is also a father of a young child and is motivated in role modelling positive health behaviors. However, MG has very little experience with using either general parental, or father specific parenting apps. MG did not have any involvement in the development of MBN app or any similar app, and this was made clear to the participants at the beginning of the focus groups and interviews. MG was conscious that some fathers will not share his knowledge and attitude toward the development of healthy behaviors and was careful not to judge participants in interviews [48].

### Ethical Considerations

This study was approved by the Deakin University Human Research Ethics Committee (HEAG-H 30_2022). All participants were provided with an Aus $25 (US $15.6) supermarket or home hardware shopping voucher for their time. Written informed consent was obtained from all fathers before participation.

## Results

### Participants

Of the 200 surveys completed in the larger study, 58 participants expressed an interest in participating in this study and were invited to take part in the focus groups and interviews. Of these, 18 indicated interest in either the initial, or one of 2 reminders to participate. After organizing time slots for focus groups and interviews, 4 of the participants cancelled their scheduled participation and did not reschedule an alternate time and were omitted from the study. A total of 5 participants chose to have an interview and 9 took part in one of 3 focus groups, which ranged from 2-5 participants. Interviews took an average of 51 (range 39-64) minutes, while focus groups lasted for an average of 58 (range 51-73) minutes.

The 14 participating fathers were on average around 40 years of age, had 1 or more children aged 5 years or younger with an average age of 2.4 years (Table 1). The majority of the fathers identified as Australian born (12/14) and all spoke English at home and were married. The majority of fathers were university educated (10/14) and were employed full-time (12/14). Participants in this study were broadly similar of survey participants; however, in contrast to participants in the survey who were 61.5% metropolitan based, the majority of fathers in the qualitative study (64.3%) were region or rural based (9/14).

Five key themes (and 19 codes) from the focus groups/ interviews relate to the previous parenting information seeking of the fathers, the functionality of the MBN app, fathers’ engagement with the MBN app, the aesthetics of the MBN app, and fathers’ perceptions of the information in the MBN app (Table 2).

**Table 1:** Characteristics of fathers participating in focus groups and interviews.

Characteristics			Fathers and children (N=14)
Father’s age (years) mean (SD), range			40.2 (5.4), 51.1-31.5
Child’s age (years) mean (SD), range			2.4 (1.9), 0.7-5.8
**Father status**			
	1 or more children aged 5 years or younger	14 (100)	
**Age of children**			
	Infant (birth to 1 year)	5 (35.7)	
	Toddler (2-3 years)	4 (28.5)	
	Preschool (4-5 years)	5 (35.7)	
**Level of education**			
	High school	1 (7.1)	
	Trade certificate/ TAFE^a^	3 (21.4)	
	University	10 (71.4)	
**Employment**			
	Full-time	12 (85.7)	
	Home duties	1 (7.1)	
	Other (share trader)	1 (7.1)	
**Marital status**			
	Married/De facto	14 (100)	
**Location**			
	Metropolitan	5 (35.7)	
	Regional/rural	9 (64.3)	
**Country of birth**			
	Australian born	12 (85.7)	
	Other	2 (14.3)	
**Language spoken at home**			
	English	14 (100)	

^a^Technical and further education.

**Table 2:** My Baby Now (MBN) app focus groups/ interview themes, codes, and illustrative quotes.

Themes and codes			Example quotes
**Previous information seeking**			
	Traditional	We’ve got a local library, so we try and go there for information about development and stuff. (F1) They [parenting groups] tend to fall back into that stereotype that moms are the primary carers. (F6)	
	App technology	I do have an app that at a certain time every day is going to send me a message and you get used to it. (F10) I've used one [an app], it did tell you what stage your baby's at, like okay, you’re at week five, so you may notice this behaviour. (F2)	
	Social media	They're absolutely great, there’s a dad's one on Facebook. I like to know what other dads find challenging or have questions about. (F9) There's a Facebook page - Advice for Dads. It became pointless, as every experience was different and just not that relevant to me. (F3)	
	Websites/YouTube	It can be stressful, just going onto a parenting website and you get bombarded with pop-up windows and ads. (F1) I want it to be accessible, so if I was cooking, you don't have to go into the app, you can just access it immediately on youTube (F14)	
**Functionality of the MBN app**			
	Ease of navigation	That's good to be able to jump around, because you’ll read something and then you'll get further down, and the reference is further away (MBN app). (F7) I understand when you're in, it went to 3 to 4 months, it just takes you to that section within a really long sheet. I started scrolling around and then I realized I wasn't looking at 3 to 4 months (MBN app). (F10)	
	Useful tools	So it’s good that it tells you foolproof. It tells you how to safely set it up [play equipment] and what you'll need. I think those kinds of things are fantastic. (F14) You look at it (MBN quiz), and go, bang, bang. You go tick, tick and then it’s done…and you get the follow-up information straight away. (F12)	
**Fathers engagement with the MBN app**			
	Tools to bond	I went to the video library, had a look at the video for chicken Bolognese. Easy to follow, the video was perfect. I was like, oh, that's easy. It’s their favourite. (F6) Having lots of different activities [in MBN] is fantastic, to be able to come up with new games and experiment with them and play with them (children) to help them create their own. (F4)	
	Customization videos	Having some presented by a male would be advantageous. You're not feeling like you're being lectured to by mums. (F11) In the recipe sections, the videos, it's all veggies and fruit. You could chuck meat on there because for dads, it’s going to resonate with him. (F2)	
	Dads’ sanctuary	To engage a dad, you need other dads already there, so they feel more comfortable, and this is actually a space for dads. (F6) Moms are always really keen if they think you're this bumbling idiot dad, and they can give you all the answers, but as soon as you have an opinion, the moms don't want you in their forum anymore. (F12)	
	Interactivity (push notifications)	That American one (different app to MBN), you can imagine it was over the top, trying to pump you up. But it was good to get those kind of positive messages (reassuring/motivational messages for dads). (F4) Age-based, just some prompts using the age, whether it was those age bands that you had earlier each month in the first 12 months and then probably less frequent after that depending how far out it goes. (F11)	
**Aesthetics of the MBN app**			
	Layout (clutter)	Subheadings or something just to, because there’s a lot there, it's quite comprehensive, but just to make it more intuitive to navigate. (F3) if you've got a few minutes to have a look at this, but then you've got to go away for something, just to be able to go back to where you were and go on with what you were doing would be great. (F7)	
	Graphics	The information’s there plus the visual video too. So, there’s a structured approach, that’s tailored to how you learn, so you can go bang. (F9) I like the pictures and stuff. That’s how I’ve always picked up stuff [retained information]. (F2)	
	Visual appeal	I know it’s probably going to be very hard, but so when you do look through a lot of pictures, there aren’t really any men in any of the photo’s. (F1) I mean, that bottom photo with the pregnancy where the mom’s in the pool laying on her back. I mean, she could be being held by her husband in the pool. Why not? (F13)	
**Fathers perceptions of information in the MBN app**			
	Specificity	It breaks the recipe up and says, at this point you take the baby's portion out and then you'll add, spices chili or whatever else, which is quite handy when you are cooking for your family. (F9) Quite a variety of topics [active play], I like that. Some apps, it just gives you a tiny bit of information and topics, but here [MBN], it puts it down for the different ages, what they should be doing. (F7)	
	Comprehensiveness	We've had issues, if a bloke takes a child into a parent's room, there’s a “what are you doing in here” attitude”. You feel rejected. it'd be good to have advice there, how dads can manage that sort of thing. (F12) Just prioritizing the topics based on the app. I think, although genders is one thing and roles. So, it could be, it's not just dad, mom, it's also - are you the primary care or secondary or what's your role? (F2)	
	Availability of info - breastfeeding	The breastfeeding thing, even if it's not as relevant for dads. It would be great if there was a section that's like how to support your partner during breastfeeding. (F2) Because in the early stages it's hard. The baby's not latching and you're just standing there useless as a dad. (F3)	
	Credibility of source	Yeah, she obviously knows what she’s on about [cooking video], but I can follow it ok. She’s explaining it ok. (F3) But, it’s like, is this all kosher? Is it what the professionals recommend [play activities]. (F10)	

### Theme 1: Previous Information Seeking

This theme included the experiences of fathers so far in searching for information in their fatherhood journey. Although all fathers were proficient using modern technology, such as smart phones, most of the fathers also made use of traditional methods, such as hard copy books when searching for information. It was apparent that most fathers did not necessarily have a preferred source of information, they did however, want information to be credible. Many fathers had also involved themselves in parenting groups but highlighted negative experiences in these groups where invariably they felt they did not belong (subtheme 1.1). The majority of fathers had made use of apps on their smart devices and had found the experiences, such as getting age specific information through reminder texts, to be positive (subtheme 1.2). The majority of fathers used social media in some form for information seeking and many highlighted the reassurance they felt in learning of the experiences of other fathers. However, other fathers reported frustration in navigating the quantity and range in quality of information on social media (subtheme 1.3). Similarly, many fathers found the convenience and accessibility of the internet in seeking information on the internet to be positive, however they could feel overwhelmed by the amount and variable credibility of information they must process (subtheme 1.4). Across all sources of traditional or modern sources of information, fathers highlighted frustration that it was targeted more to mothers than fathers.

### Theme 2: Functionality of the My Baby Now App

In this theme, fathers’ spoke about what was important to them in regard to how the MBN app should function. Most fathers made comments about the importance of being able to navigate around the MBN app easily. However, some fathers highlighted some frustration with needing to scroll or swipe excessively, which resulted in them losing their place (subtheme 2.1). When able to locate and engage with the sections of the MBN app as desired, fathers spoke positively about the reassurance they felt from the information provided (subtheme 2.2).

### Theme 3: Engagement With the My Baby Now App

In this theme, fathers spoke about the reasons why they may engage with the MBN app. Many fathers spoke positively about the combination of written and video information available, which provided ideas and strategies about how to engage with their children in areas such as pleasurable games and providing enjoyable food (subtheme 3.1). A consistent theme from fathers was for them to engage with the MBN app, it needs male imagery that could also include males presenting information in videos (subtheme 3.2). Fathers also highlighted the need for information in areas (such as male-only forums) where they could get information and exchange viewpoints without the threat of feeling out of place (subtheme 3.3). Fathers spoke positively about getting push notifications delivered to their smart devices, including specific health-related information about their child and simple messages of encouragement. However, the majority of fathers highlighted that daily push notifications would be too excessive, with some fathers preferring weekly or monthly notifications as the child ages (subtheme 3.4).

### Theme 4: Aesthetics of the My Baby Now App

This theme provided fathers the opportunity to highlight how the presentation of the MBN app would affect their attraction and potential use of the app. Some fathers found that information in the MBN app can appear cluttered due to the amount of information and the lack of defined sections, such as information presented by age or stages of development. A number of fathers stated that this could be remedied by the use of more subheadings (subtheme 4.1). In receiving information, fathers found the use of graphics and videos to explain information could facilitate a positive learning experience (subtheme 4.2). However, most fathers had negative perceptions about the distinct lack of visual images of fathers and lack of pictures that fathers would be interested in, such as more meat-based meals (subtheme 4.3).

### Theme 5: Perceptions of the Information in the My Baby Now App

When discussing the type of information that they would like to see, fathers consistently made comments that they were appreciative of specific, relevant information that provided them with clear steps to follow, such as age-appropriate play activities and recipes (subtheme 5.1). However, many fathers also highlighted the need to have information in different areas of fatherhood that they were unfamiliar with, such as tips on the social etiquette of using baby change rooms and tips on food storage (subtheme 5.2). The majority of fathers highlighted that helping with the demands of breastfeeding was a constant source of stress and some thought a specific section for fathers and breastfeeding would be advantageous (subtheme 5.3). Fathers highlighted the need for factual information, with some fathers stating that they did not mind if a male or female presented information in videos, just as long as it was factual (subtheme 5.4).

## Discussion

### Principal Findings

This study adds to a limited pool of research that uses qualitative enquiry to gain fathers perspectives on how parenting apps can be improved to be more engaging for fathers [24]. From conducting focus groups and interviews with fathers, 5 themes emerged; previous parenting information seeking, functionality of the MBN app, fathers’ engagement with the MBN app, the aesthetics of the MBN app, and fathers’ perceptions of the information in the MBN app. The information gained from fathers about mHealth technology is important, as traditional and typical contemporary domains for fathers to find information on parenting, such as social media and parenting websites have typically targeted mothers, with few father-specific resources [49]. Indeed, some parenting apps to date have portrayed a dismissive attitude to fathers’ roles and experiences and what they can contribute as a parent to children in early stages of development [28,50].

Findings from our study revealed that fathers have previously used a variety of traditional and contemporary sources of information, including books, social media, parenting groups, apps, with parenting websites and parenting material on YouTube (Google) being the most prevalent. This is in line with previous Australian research, which also found the web-based activities was the main resource fathers used to find information on their child’s health, for reasons of convenience and speed, and this was particularly so in fathers of a higher socioeconomic position (SEP) [51]. Our sample consisted mostly of higher SEP fathers (by education) with over 70% of fathers being university-educated, and the preferences for internet usage highlighted by fathers is in line with a “digital divide” noted by Laws et al [51], whereby higher educated fathers are more likely to use parenting website activities for seeking information on children’s health. Understanding differences in mHealth app usage by fathers seeking children’s health information, delineated by SEP, remains unclear and will benefit from further research.

Despite being the prominent choice, seeking information through parenting websites and YouTube was widely reported to be a frustrating endeavor for fathers in our study, whereby many highlighted they could not locate what they desired due to being overwhelmed by excessive information of questionable quality. This led to some fathers having a negative perception of internet-based parenting information and resources and avoiding its use [52]. Similar findings were reported in the Australian qualitative study of Walsh et al [10], whereby fathers highlighted a desire to be better informed about their young children’s diet and physical activity needs, but felt there was a lack of useable and credible information to guide them. Of fathers who had previously used apps in our study, not only was the convenience of the app in providing prompt information important, but also the value of an app in providing useful and credible information in the one area.

Many fathers in our study highlighted their negative perception of parenting information or imagery encountered previously, and also in the MBN app, that included only maternal content. Such perceptions are important considerations for the engagement of fathers, as it has been found that representations and languages that embed social norms of fathers not being involved in parenting duties, have led to paternal disengagement from parenting programs [53]. For fathers to be motivated to participate in parenting research and programs, research has highlighted the need for stratification, with fathers specifically named and targeted to garner paternal interest [54]. This need was present in the recommendations from fathers to improve the MBN, whereby it was unanimously highlighted that the MBN app needs greater father-specific information and imagery to appeal to and engage with fathers.

Further to the requests for greater father imagery and content, fathers also widely requested for there to be father-only forums. Evidence suggests that fathers engage with other fathers in a different style than mothers do, with more humor and less formality in their social media and chatroom interactions [55]. Their preference for male-only domains in apps may provide them with an opportunity to discuss sensitive matters while maintaining a perception of control through the use of humor while on an equal footing to other fathers [56]. Canadian research into engaging young fathers in parenting programs reveals that they will be more likely to engage if programs initially involve informal meetings in places of comfort with their male peers, such as sports fields [23]. This allows fathers to seek help in an environment of perceived security without looking “weak,” as experienced in more formal settings such as traditional health care [55]. It was interesting to note in our focus groups and interviews, how positively and openly fathers engaged with each other, with regular humor evident, in discussing sensitive developmental issues without previous contact with each other. Another potential benefit of men-only forums is that fathers who have had knowledge and previous experience with other children may be able to pass this knowledge onto younger or less experienced fathers who benefit from guidance [57].

Well-designed tools available through smart technology have the potential to provide fathers with the information they want [15,58], and fathers in our study were mostly able to navigate through app technology when seeking information. Similar to previous research [23,28], the fathers in our study reported that when they were able to find information, they could understand on the MBN app, and improved confidence and enjoyment was noted in their engagement with their child. Receiving notifications through smart phone technology has proven to be beneficial for mothers as well as fathers in gaining information [59,60], and fathers in our study were mostly positive about receiving push notifications from the MBN app, particularly in areas of low knowledge, such as breastfeeding. Push notifications, therefore present as a valuable strategy to deliver desired breastfeeding information to fathers wanting to be supportive, which is vital for mothers to facilitate the demands of breastfeeding [61]. Similar to Australian research into the effects of supportive text messages on new fathers’ mental health [15], many of the fathers in our study expressed a belief that messages of support would be beneficial in managing parental pressures and lowering isolation. The pressures of raising young children are a stressful time where new demands are being put on parents [62], and many fathers in our study highlighted the significant impact of feeling useless and isolated had on their mood. In demanding times, if reassurance and guidance are not provided by others, mHealth technology presents as a valuable resource for fathers to use in their parenting journey.

While this paper highlighted the potential benefits of providing fathers with credible, evidence-based mHealth resources, it did not have the scope to explore the feasibility of developing and maintaining a usable app for fathers to use in their everyday lives. Recent American evidence suggests that it can take over 6 months and costs approximately US $ 270,000 for the development of an app, with ongoing maintenance costs an additional expense [63]. It was noteworthy in this study, that rather than advocating the need for father only apps, many fathers instead highlighted their desire to see father specific sections in current parenting apps. By having father specific sections in current parenting apps, the desired resources that fathers have raised in this study, such as positive male imagery, father only forums, and tailored push notifications, could be provided without the financial burden that a stand-alone app would encumber.

### Strengths and Limitations

This study has a number of strengths and limitations. A key strength was the qualitative design, which allowed fathers’ perspectives to be explored in more detail through focus groups and interviews, thereby providing a deeper understanding of fathers’ experiences and viewpoints. The results of this paper provide qualitative evidence from fathers about what is needed in an mHealth resource for them to engage with it and find informative and useful. This is important for the promotion of coparenting, as more traditional methods of providing education to fathers, such as parenting classes, have not been an effective way to provide support fathers in paid employment [15]. Coparenting research has advocated the need for modern technology such as mHealth, to reach parents during the demanding postpartum period to promote greater coparticipation in the promotion of positive health behaviors [62]. Such co-operative parenting behaviors have in turn been shown to lead to such benefits as improving the child’s obesity status [4] and the social-emotional well-being of the child [64,65].

In line with noted recruitment difficulties in existing paternal research [17,29,54], the recruitment of a desired number and varied sample of participants proved difficult for this study. In addition, the sample was limited by fathers being mostly higher educated, predominantly Australian-born, who by their interest in this study, were likely to be health conscious and committed fathers. While this may have contributed to social desirability bias in their responses, the fathers in our study were open and forthcoming with divulging their challenges and offered valuable insight and recommendations for improvement of the MBN app. Future research will benefit from the inclusion of a varied sample of participants, including fathers of low SEP and the inclusion of fathers of differing family status, such as single fathers and same sex couples. In addition, as this paper was predominantly made up of a culturally homogenous sample of participants, caution will need to be exercised in extrapolating the results internationally, as approaches and beliefs about fathering varies considerably across cultures [8].

Finally, many fathers were able to make themselves available only at select times, which precluded them from taking part in focus groups with other fathers, and instead took part in a one-on-one interview with a researcher. This denied some fathers the opportunity to engage with other fathers in a positive group setting and may have limited their opportunity to develop their views and opinions through group dynamic. The researchers do not believe that the quality of the information elicited in interviews lacked any of the richness of focus groups.

This qualitative study has provided unique insights from fathers in focus groups and interviews about how an mHealth parenting resource, in the form of app technology, can be improved to enhance the engagement of fathers in early childhood. While current parenting apps, such as MBN, have proved advantageous in delivering factual information to fathers expediently, the information can fail to appeal to the specific needs and wants of fathers. Fathers committed to the promotion of positive health behaviors to their children risk becoming frustrated and potentially disengaged if father-specific material is not provided. Through the promotion of father specific information, use of positive imagery and descriptives of fathers in their role, promoting father exclusive domains such as forums, and using positive reinforcements such as push notifications to support and give guidance to fathers in an area of uncertainty for them, app technology can be a valuable resource for fathers in their parenting journey.

## Appendix

Multimedia Appendix 1 Additional information.

## Abbreviations

INFANT: Infant Feeding Activity and Nutrition Program
MBN: My Baby Now
mHealth: mobile health
SEP: socioeconomic position
uMars: end user version of the mobile rating scale

## Data Availability

The datasets used and/or analyzed during this study are available from the corresponding author on reasonable request.
