# Supplementary material for: Practicality of the My Baby Now App for Fathers by Fathers: Qualitative Case Study
Source: JMIR Pediatr Parent. 2025 Feb 21;8:e64171. doi: 10.2196/64171 (PMC11890138; doi:10.2196/64171)
Supplement: Multimedia Appendix 1 [file pediatrics_v8i1e64171_app1.docx]

**Supplementary File 1:**

**Introductions (5 - 10 Minutes)**

- **Power point 1 -** Thank you for all joining me here today. For those who are joining me for the first time and haven’t taken part in study 1, welcome to the Engaging Dads Study at Deakin University. Also, welcome to the returning dads who took part in study 1 and are joining me again to offer their valuable views and experiences in the next stage. The overall aim of this and the other studies that make up my PhD is to investigate how to support dads in helping their children learn to eat well and be active. While the overall study looks at early childhood, for the study 2 focus group which you are all involved in today, I will be looking at the period from birth of 18 months. Some of you may have a child/ ren a little older than that, but that’s okay as you will still have valuable knowledge and experiences of that developmental stage. All up, I will working with approximately 20-25 other dads who will work together with myself to see if there are ways that the current MyBabyNow app can be improved to attract and be useful to dads such as yourself.
- **Power point 2** - Can I get you all to quickly introduce yourselves, where your from and number + ages of children.
- Thanks. I just wanted to emphasise that these focus groups are going to be run in the spirit of mutual contribution between myself and all the dads here today. I really want to hear about your unique opinions and experiences as they are all valuable to this study and can help design tools and strategies to help other dads in the future to make the fatherhood journey less stressful and more enjoyable.
- **Power point 3 -** We are going to be getting your thoughts and feedback working on an app today called – MyBabyNow which was developed by the INFANT team at Deakin Uni. I just wanted to quickly highlight that even the app is from Deakin Uni, its current design or its development so far is something that I haven’t been involved in, so we are all looking at the app from the same level. Two of the main purposes of the app are to -
  - Provides credible and practical advice, tips and tools to help feed your baby – whether this is breast or formula feeding, mixed feeding or introducing solids.
  - It also helps you understand your baby’s development week-by-week throughout pregnancy and until your baby reaches 12 months, providing lots of ideas for play.
  - Mainly used by mothers and we would like to make it more accessible for dads.
- **Power point 4 –**
- So the goals for today will be to –
  - Look at the existing resources in the solids, feeding and play section of the MyBabyNow app and as a group discuss their effectiveness and think about ways they could be changed to be more applicable to dads.
- Can I just confirm that everyone has got my message and has had an opportunity to download the app to their mobile phone? If no, I have put the link into the chat function. Can I ask you to download the app now, which should take about 5 minutes to be up and running.
- For those who have already downloaded the app, you can take some minutes to have a play with the different parts of the app while the others download the app to their phones. We will be looking specifically at the solids, recipes and play topics as a group once everyone is ready.
- Ok and for those who have now got the app downloaded, I will also now ask everyone to have a browse at the specific topics - specially the solids, recipes and play topics. When your finished, can I ask you to do 1 of the quizzes about solids or play in the activity section of the app.

**Experience bank discussion (10 mins) –**

*Goal is to quickly unload people’s experiences and avoid focusing on negative experiences during the rest of the focus group.*

- It would be great to hear about your experiences so far in your fatherhood journey. Firstly, can I hear about your experiences when your child started solid foods.
- [General feedback sought – good / bad? Leading to more specific questions - ]
- How involved were you?
- what did you want to know?
- where did you go for information/support?
- what did you find most challenging?

It would be great to hear about your experiences of trying to encourage some active play with your baby, starting with tummy time when they were a young baby to spending time outdoors and exploring their environment as they became more mobile. I am also interested in hearing your thoughts about screen time such as watching TVs, tablets or phones which are of course everywhere these days.

[General feedback sought – good / bad? Leading to more specific questions - ]

- How involved were you in playing with your baby or managing screen time?
- what did you want to know?
- where did you go for information/support?
- what did you find most challenging?

I just wanted to talk further where you all went for information/ support. Some of you (or none?) mentioned that you used apps. What apps did you use? I just wanted to ask how you came across these apps –

- recommendations from friends / family?
- health professionals? Etc
- googling?
- What did you like about them?
- What particular features did you like and interested? For those who haven’t used app ‘what features or information do you imagine you would like in an app’
- How do these compare with the resources / strategies on the MyBabyNow app?

**Divergence (20 – 25 mins)**

Use of HMW questions.

Now let’s look at My Baby Now app and get your feedback

Solid Foods -

*Facilitator will share articles (by zoom ‘share screen’ option)*

- Can I ask everyone to go to topics and open the solids topic.
- Have a browse through the articles in this topic.
- Can I ask you to open one article of your choice and have a quick browse of the article.
- Now I would like to get your feedback. What do you think about the types of articles covered in the solids topic? Does it cover what you need to know or is there things missing?
- How useful did you find the article you read?
- What did you think about how the information was presented?
- How understandable was it?
- Facilitator finally uses ‘HMW’ (How Might We) –
  - Improve the information provided in the MyBabyNow app about introduction of solid food

Recipes -

*Facilitator will share articles (by zoom ‘share screen’ option)*

- Can I ask everyone to go to topics and open the recipes section.
- Have a browse through the recipes.
- Can I ask you to open one recipe of your choice and have a quick browse of the recipe.
- Now I would like to get your feedback.
- What do you think about the layout of the recipes section?
- Do you like video section? Are you able to follow the instructions.
- By reading / viewing material in the recipe’s section, do you have more confidence to try something that you otherwise would have thought was too hard / complex?
- Can you imagine yourself using the recipe section of the app?
- Facilitator finally uses ‘HMW’ (How Might We) –
  - Improve the information provided in the MyBabyNow app about recipes for children < 18 months.

Play -

*Facilitator will share articles (by zoom ‘share screen’ option)*

- Can I ask everyone to go to topics and open the play activity section.
- Have a browse through the articles in this topic.
- Can I ask you to open one article of your choice and have a quick browse of the article.
- Now I would like to get your feedback. What do you think about the types of articles covered in the play section? Does it cover what you need to know or is there things missing?
- How useful did you find the article you read?
- What did you think about how the information was presented?
- How understandable was it?
- Facilitator finally uses ‘HMW’ (How Might We) –
  - Improve the information provided in the MyBabyNow app about play for children < 18 months.

Push notifications / messages.

- You may have seen / received some of the messages from the MyBabyNow app. I am going to show you some messages from the solids and play sections of the app, and then I will get you to vote on the usefulness of this message via menti.com either using your phone or your computer. All messages will have 5 options to choose from – very helpful, helpful, neutral, unhelpful and very unhelpful. Let’s vote on these messages in the solids section -
- Wondering how ‘child’s’ diet aligns with dietary recommendations for toddlers? Take our quiz here.
- Is child still saying ‘no’ to some vegetables, don’t give up! Remember it can take 15 or more tries before ‘child’ learns to like them.
- Toddlers often want to ‘do it themselves’, and this applies to eating too. Worried about ‘child’ choking? We have some information that will help!
- Children are healthiest when parents provide, and kids decide. Learn more about feeding roles here.
- Keen to eat more healthily but concerned about the cost? Check out money savings tips here.

*Each dad votes on each of the above messages. Facilitator will discuss answers with dads. Why / why not? So how can this be improved?*

Ok, now let’s vote on these messages in the play section -

- Worried that ‘child’ will be bored if they are not allowed to use screens? Learn about the importance of independent play here.
- Looking for ideas to keep ‘child’ occupied? We have some ideas that don’t involve screens here.
- Check out our tips on creating safe spaces for ‘child’ to play independently here.
- Is ‘child’ meeting the physical activity recommendations for 12-month old’s? Find out here.
- Now that ‘child’ is gaining some more movement skills, consider creating a more challenging obstacle course. Learn more.

*Each dad votes on each of the above messages. Facilitator will discuss answers with dads. Why / why not? So how can this be improved?*

*- Facilitator will further seek dads preferences for how many messages per week and whether there is a preference for SMS or Push notifications.*

Quizzes -

- You all completed a quiz at the start of todays focus group. Can I ask your thoughts about the usefulness of quizzes or any of the others that you may have completed previously within the activities section of the app?
- How could these be improved / be ade for relevant for your needs?

Forum section of app

*Facilitator will invite dads to browse the forum section of app.*

- What do you think about having a forum in the app where you can ask questions or share experiences with other parents?
- What are your preferences for being involved in male only forums, or dad & mum forums? Do you prefer dads only? If so, why? Have any dads made use of dad only support groups – like beers and bubs?

Any Other suggestions:

Dads invited to offer any other thoughts / suggestions. Any other suggestions on how to improve the look/feel or other aspects of the My BabyNowApp to make it more appealing for Dads.

Specific points for discussion –

- How could the look or feel of the app be made more appealing for Dads?

- What strategies / resources on the app will help fathers engage (become involved).

-What strategies / resources on the app will give fathers greater confidence?

During the divergence process, each member presents their ideas while the rest of the team takes note of –

- What ‘I like’ – what you like about the solutions and
- ‘I wonder’ – how you might improve the solutions.

**Convergence (10 minutes)**

Converging is a qualitative process. *For each of the above sections, facilitator will provide a brief overview / feedback on the main points that have been raised as to how the app can be improved / refined. The group will be asked for any final thoughts on each section.*

*Dads will finally be asked if they could imagine themselves using an app like this long term? Why or Why not? What would encourage Dads to use an app like this. For example, would it matter who recommended the app e.g. health professional/maternal and child health nurses, their partner/wife, or if they just stumbled across it on social media? If needed, a vote will be conducted to consolidate the best designs into one solution that they would like to see in the MybabyNow app.*

**Final points for discussion – (10 minutes)**

At conclusion of workshop, researcher will summarise meeting and the resources / strategies that have been agreed upon. Participants will be advised that the results of the focus group will be provided to the app developers for consideration of possible alterations. Fathers may then be contacted in future to provide their opinions / comments on the revised app.
